# Urban-resolved scales amplifies torrential rainfall in coastal megacities

**DOI:** 10.1038/s41598-025-23906-y

**Published:** 2025-11-17

**Authors:** Konduru Rakesh Teja, Rahul Bale, Anu Gupta

**Affiliations:** 1 Data Assimilation Research Team, RIKEN Center for Computational Science, Kobe, Japan; 2 Complex Phenomenon Unified Simulation Research Team, RIKEN Center for Computational Science, Kobe, Japan; 3 Graduate School of System Informatics, Kobe University, Kobe, Japan; 4 Graduate School of Information Science, University of Hyogo, Kobe, Japan; 5 Present Address: Earth Observation Research Center, First Space Technology Directorate , Japan Aerospace Exploration Agency , Tsukuba Space Center, Sengen 1-1, Tsukuba, Ibaraki Japan

**Keywords:** Climate sciences, Atmospheric science

## Abstract

This study unveils the ability of 200-m near to Large-Eddy Simulation (LES) to resolve smaller scales in urban areas of a coastal megacity Chennai in India, which are crucial for accurately modelling Urban Induced Turbulence (UIT) and Urban Heat Island (UHI) effects. Unlike physics-based ensemble (PBE) models in kilometer-scale simulations, LES captures fine-scale atmospheric dynamics, offering enhanced resolution to simulate localized turbulence and heat flux variations. With a resolution of 200 m, Weather Research Forecasting (WRF) LES simulation can convincingly resolve a higher urban heat flux, friction velocity, surface wind speeds, and moisture transport over urban areas. These factors are found to play a critical role in intensifying extreme weather events such as heavy rainfall, especially in the downwind regions of coastal megacities like Chennai. This phenomenon is driven by LES-resolved interaction between the urban heat island effect and the prevailing winds.

**Supplementary Information:**

The online version contains supplementary material available at 10.1038/s41598-025-23906-y.

## Introduction

Urban environments are increasingly at risk from extreme weather events due to the combined pressures of climate change and rapid urbanization. These events can result in significant economic and social disruptions, necessitating improved prediction and understanding of urban meteorological processes. Traditional weather prediction models, such as those based on mesoscale meteorology, often operate at relatively coarse resolutions, typically on the order of kilometers. These models are limited in their ability to resolve the complex and heterogeneous nature of urban areas, leading to inaccuracies in simulating phenomena like heatwaves, storm surges, and urban flooding. Large Eddy Simulation (LES) is a powerful tool for overcoming these limitations by providing high-resolution, three-dimensional representations of atmospheric processes, capturing the interactions between urban structures, vegetation, and the atmosphere with remarkable detail^1–3^.

Physics-based models operating at kilometer-scale resolutions^4^ captures large-scale atmospheric dynamics, and they face significant challenges when applied to urban environments. The complex geometry and heterogeneity of urban landscapes introduce localized microclimates and turbulence that are not well-represented in these models. For example, kilometer-scale models often oversimplify the effects of buildings and other urban features, leading to errors in predicting temperature distributions, wind fields, and pollutant dispersion^5,6^. LES offers a solution by providing the spatial resolution necessary to explicitly simulate urban microclimates, including the effects of surface roughness, building-induced turbulence (BIT), and the urban heat island effect^7–9^.

Previous research in urban climate modeling has often focused on mesoscale models, which are suitable for broader weather patterns but lack the resolution necessary for simulating intricate urban microclimates. While these models, such as the Weather Research and Forecasting (WRF) model^10^, have been widely used to study urban climates, they often require parameterizations that can introduce uncertainties^11,12^. LES, by contrast, operates at a much finer scale, often on the order of 100–300 meters, allowing for the explicit simulation of turbulence and the detailed representation of urban features^13,14^. Studies have demonstrated that LES can significantly enhance the accuracy of urban climate predictions, improving our understanding of urban wind patterns, temperature distributions, and pollutant dispersion^15,16^. Notable work by has demonstrated the advantages of LES in capturing the fine-scale interactions between urban structures and atmospheric processes^17^, providing more reliable weather forecasts and insights into the impact of urbanization on climate.

By employing LES in urban extreme weather simulations, we anticipate gaining a deeper understanding of the localized impacts of extreme events on urban environments. The fine-scale resolution of LES allows for detailed modeling of urban features, such as buildings and streets, which significantly influence microclimatic conditions. One of the key phenomena that LES can accurately simulate is Urban Induced Turbulence (UIT), which is critical in shaping the dispersion of heat and pollutants within a city^18,19^. Compared to physics ensemble models, which average out many of these finer details, LES provides a more accurate representation of urban dynamics.

**Fig. 1:**
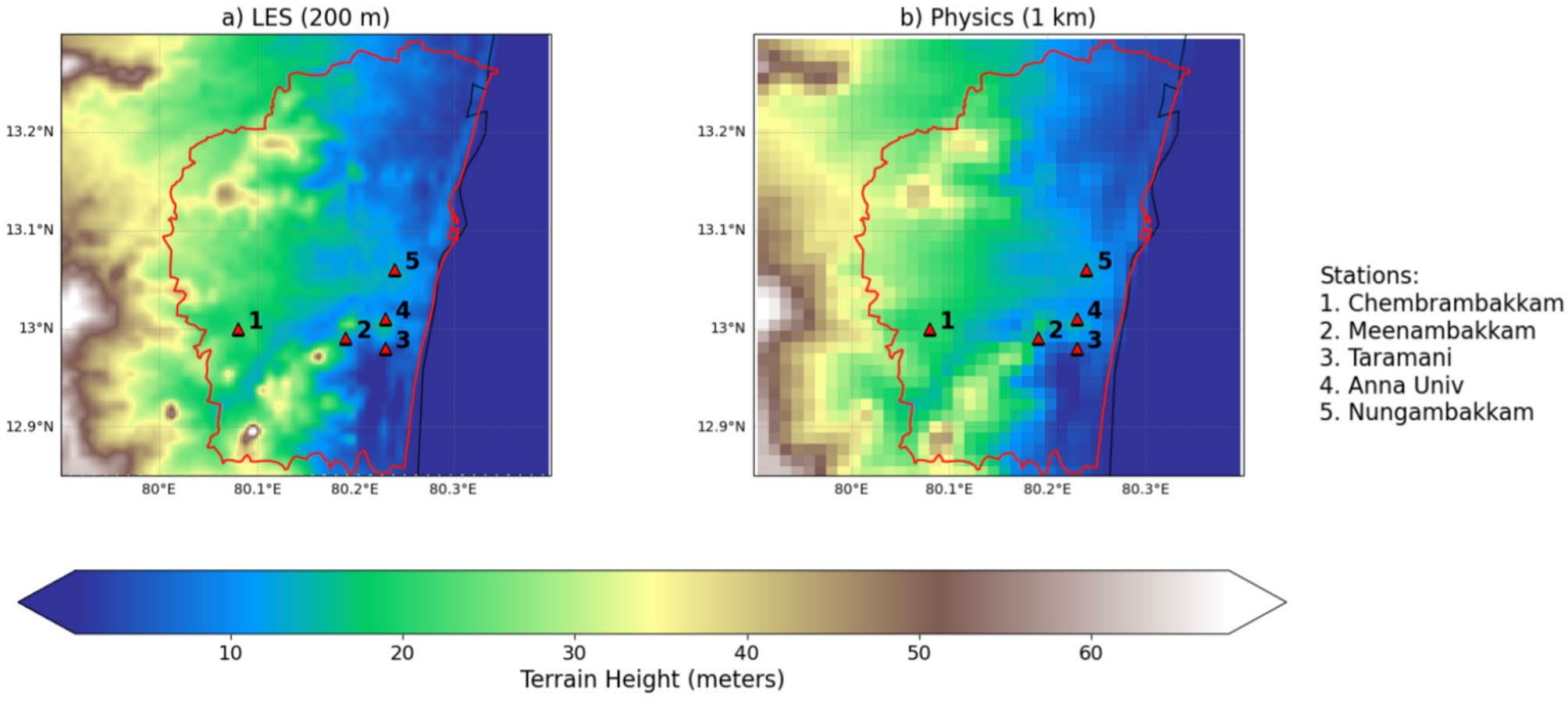
Topography (m) map over Chennai and its neighborhood as represented in the (**a**) LES (200 m) model and in (**b**) physics ensembles (1 km). Topography data in WRF model is taken from Shuttle Radar Topography Mission 3 second (SRTM 3 s) dataset.

The current study aims to explores the impact of urbanized coastal metropolitan on the extreme rainfall dynamics using the LES. The benefits of topography resolution (Figure 1) on urban extreme weather simulation representation compared between LES at 200 m to physics ensemble models at kilometer-scale resolutions. By conducting a comparative analysis, we seek to quantify the improvements in predictive capabilities offered by LES, particularly in urban settings characterized by complex geometries and heterogeneities. This study will involve high-resolution simulations of urban areas experiencing extreme weather events, such as storms, and comparing the results with those obtained from traditional mesoscale physics ensemble models^20,21^.

## Results

### LES and Physics ensemble accumulated rainfall evaluation

**Fig. 2.**
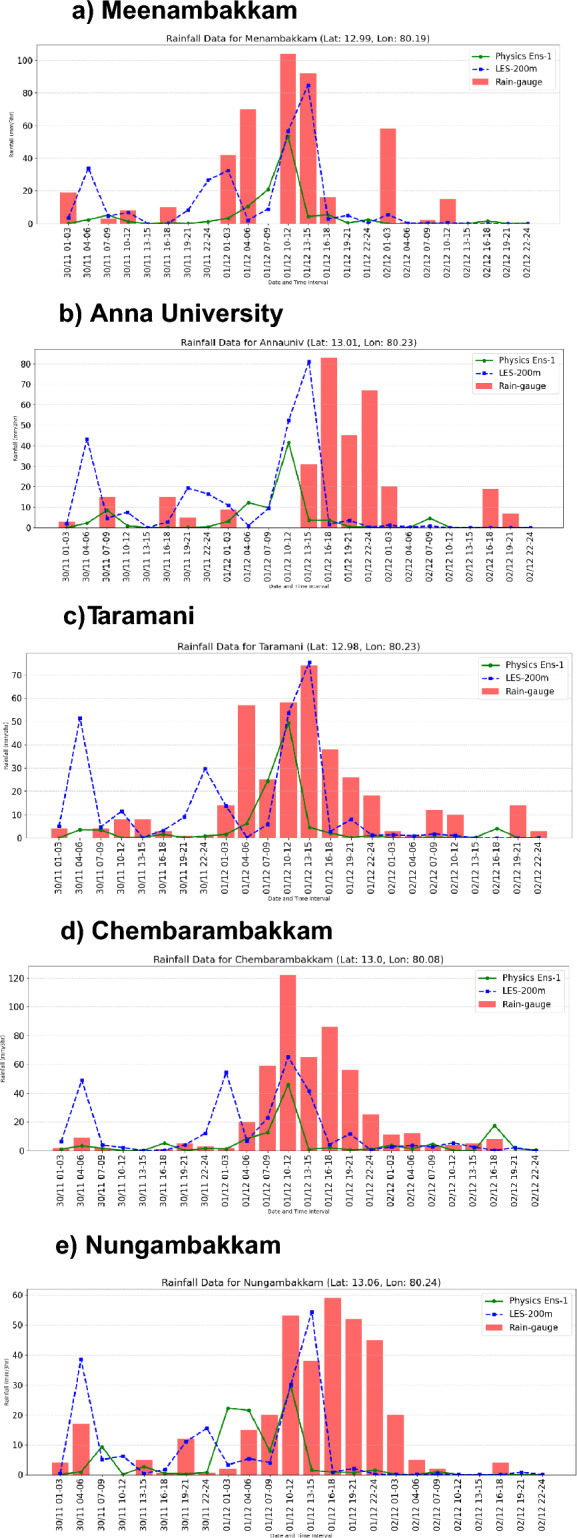
Evaluation of model simulated rainfall (mm) with the rain gauges in the Chennai metropolitan area. Observed rainfall from rain gauge, and simulated rainfall from LES (200 m), and Physics (1 km) are shown as red bar chart, blue dash line, and green solid line respectively.

In the earlier study^22^ current model simulation for 1 km was evaluated and we showed the spatial and temporal accuracy of the physics simulation (1 km) during the 2015 Chennai flood event. Further to evaluate the fine temporal and spatial distribution od precipitation from LES and physics 1 km simulation, we compared it against rain gauge observational data (Figure 2). Locations of rain-gauge are shown in the figure 1. As from this evaluation of LES (200 m) and Physics (1-km) simulation shows clear temporal variations at each grid. The LES model was evaluated using rain gauge observations across five locations: Meenambakkam, Anna University, Taramani, Chembarambakkam, and Nungambakkam. The results show that LES (200m resolution) consistently matches the observed rainfall better than the Physics ensemble. In each case, the LES output closely follows the timing and intensity of rain gauge data, while the Physics ensemble tends to underestimate or misalign with actual rainfall. LES mimics the rain-gauge diurnal variation in rainfall and captures peak rainfall at most of the locations. This suggests that LES provides a more accurate representation of localized rainfall events. The comparison highlights LES as a reliable tool for high-resolution rainfall simulation, especially in urban and coastal regions.

**Fig. 3:**
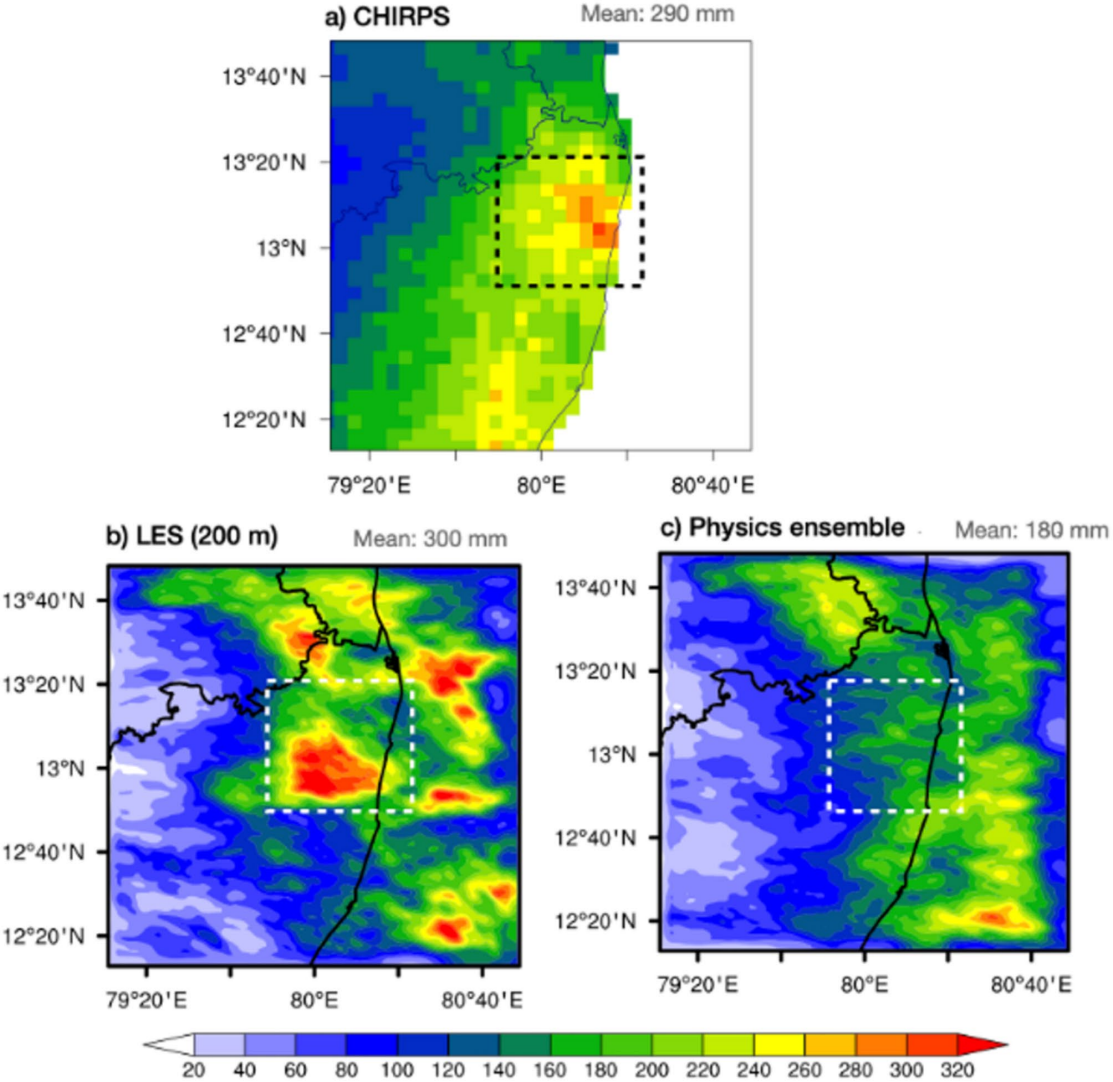
Accumulated precipitation (mm) over Chennai in (**a**) CHIRPS observations, (**b**) LES simulation, and (**c**) Physics ensemble mean for the period November 30 to December 1, 2015. Dash rectangle shows Chennai region of southeast India.

The figure 3 compares accumulated precipitation for the period November 30 to December 3 over Chennai from CHIRPS observations, LES at 200 m resolution, and a physics ensemble simulation. The CHIRPS data shows a mean precipitation of around 290 mm with distinct regions of intense rainfall spatial variability as explained by normalized standard deviation of 1. The LES simulation captures these patterns well which we confirmed by its pattern correlation of 0.50 (0.29 for Physics ensemble-1) with observation, with a mean precipitation of 300 mm and detailed spatial variability as explained by a 0.62 normalized standard deviation (0.22 for Physics ensemble-1) This reflects LES models ability to capture localized convective systems. Though precipitation magnitude is higher and comparable with CHIRPS observations, LES simulation exhibit displacement errors that can cause double penalty problem in evaluating precipitation forecast. In contrast, the physics ensemble significantly underestimates precipitation, with a mean of 180 mm, displaying a more uniform distribution that lacks the localized intensity seen in observations. This suggests that the LES provides a more spatial representation of precipitation patterns compared to the physics ensembles (see supplementary Fig. S2), likely due to its higher resolution and ability to simulate finer-scale atmospheric dynamics. In contrast, the physics ensemble, with its coarser resolution, underestimates precipitation and fails to capture the localized intensity of rainfall, highlighting its limitations in resolving the fine-scale atmospheric dynamics critical for urban regions.

### Dynamical differences in LES and physics ensemble

**Fig. 4:**
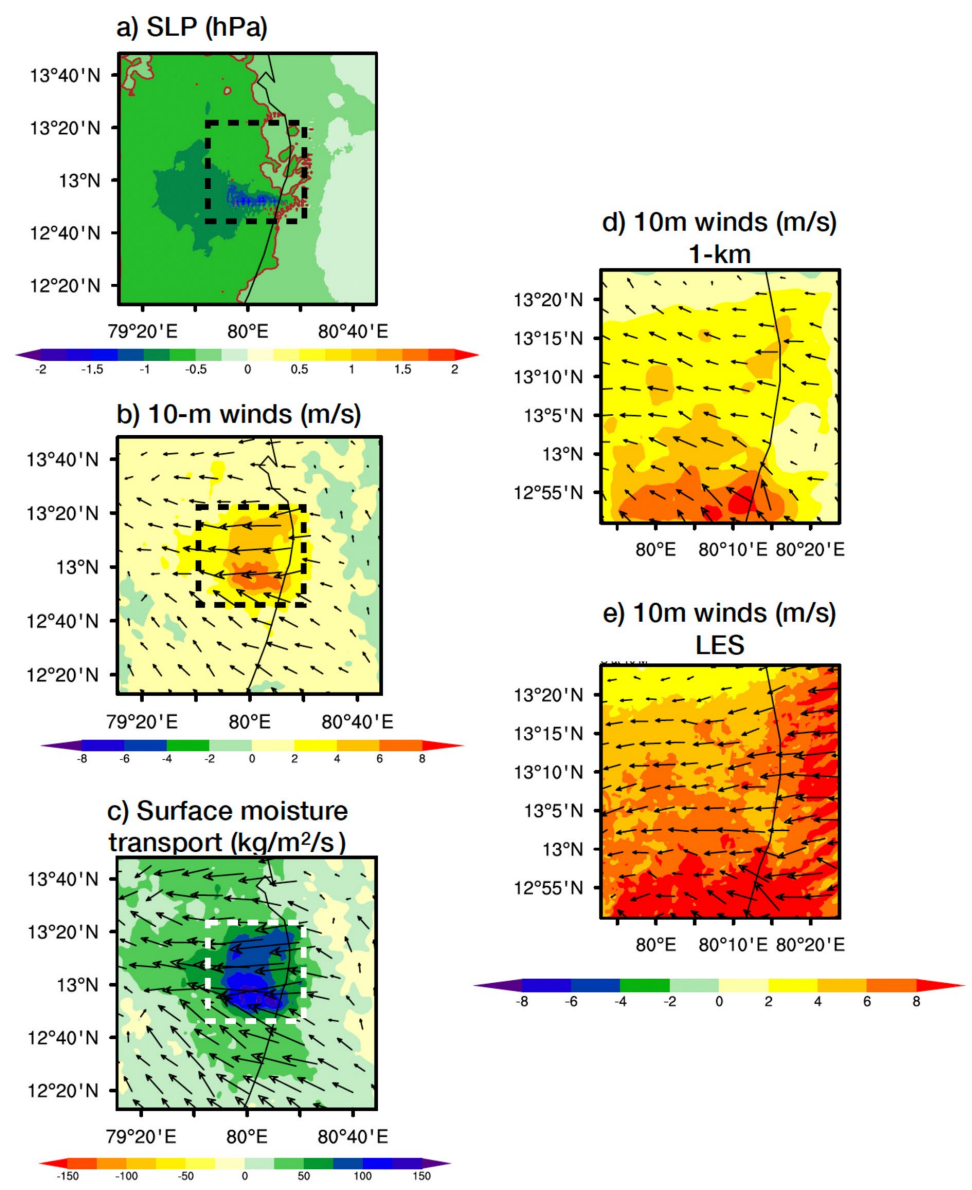
Differences in the LES simulation and physics ensemble before the heavy rainfall for (**a**) sea level pressure (SLP; hPa), (**b**) 10-m winds (m/s), and (**c**) surface moisture transport (kg/m^2^/s). Differences in the 10 m (m/s) winds of LES simulation and physics ensemble zoomed over the Chennai city region for (**d**) 1-km scale parent domain and (**e**) LES. Box shown in (**a**-**c**) black/white dash line points Chennai city region. Reference vector for winds is 5 m/s and for surface moisture transport 100 kg/m^2^/s. Surface moisture transport and winds direction shown by the vectors and their magnitude by the length of the of the arrow and filled color.

We showed differences in dynamics of LES and physics ensemble mean interpolated to the LES resolution (200 m) during the heavy rainfall event over the Chennai city of South India. Figure 4a shows difference in sea level pressure (SLP; hPa) highlighting much lower SLP (−2 hPa) in LES simulations than physics towards the south and western regions of the Chennai city. Over same region 10 m winds are 6–8 m/s higher in LES than physics ensemble showing major concentration of the surface to the lower atmosphere winds. As compared to the suburbs (outside of box in Fig. 4b), surface winds are stronger all over the city regions. Further analyzed surface wind speeds zoomed over the Chennai city as shown in the box (Fig. 4b) for 1-km parent domain simulation (Fig. 4d) and LES (Fig. 4e). LES simulation shows major heterogenous nature and intensification of surface winds only over some locations, such minute changes in the winds can be attributed to the Urban Induced Turbulence (UIT).This kind of dynamic situation can attract more moisture around >100 kg/m^2^/s (Fig. 4c) towards the Chennai city in the lower atmosphere from the adjacent sea Bay of Bengal. Similar to SLP and surface winds moisture as well attracted towards the southern regions of the Chennai city. Such higher wind speeds situation and UIT contribution may not be noticed in the 1-kilometer scale simulation of physics ensemble.

### Contribution of Urban Induced Turbulence (UIT) and Urban Heat Island (UHI)

**Fig. 5:**
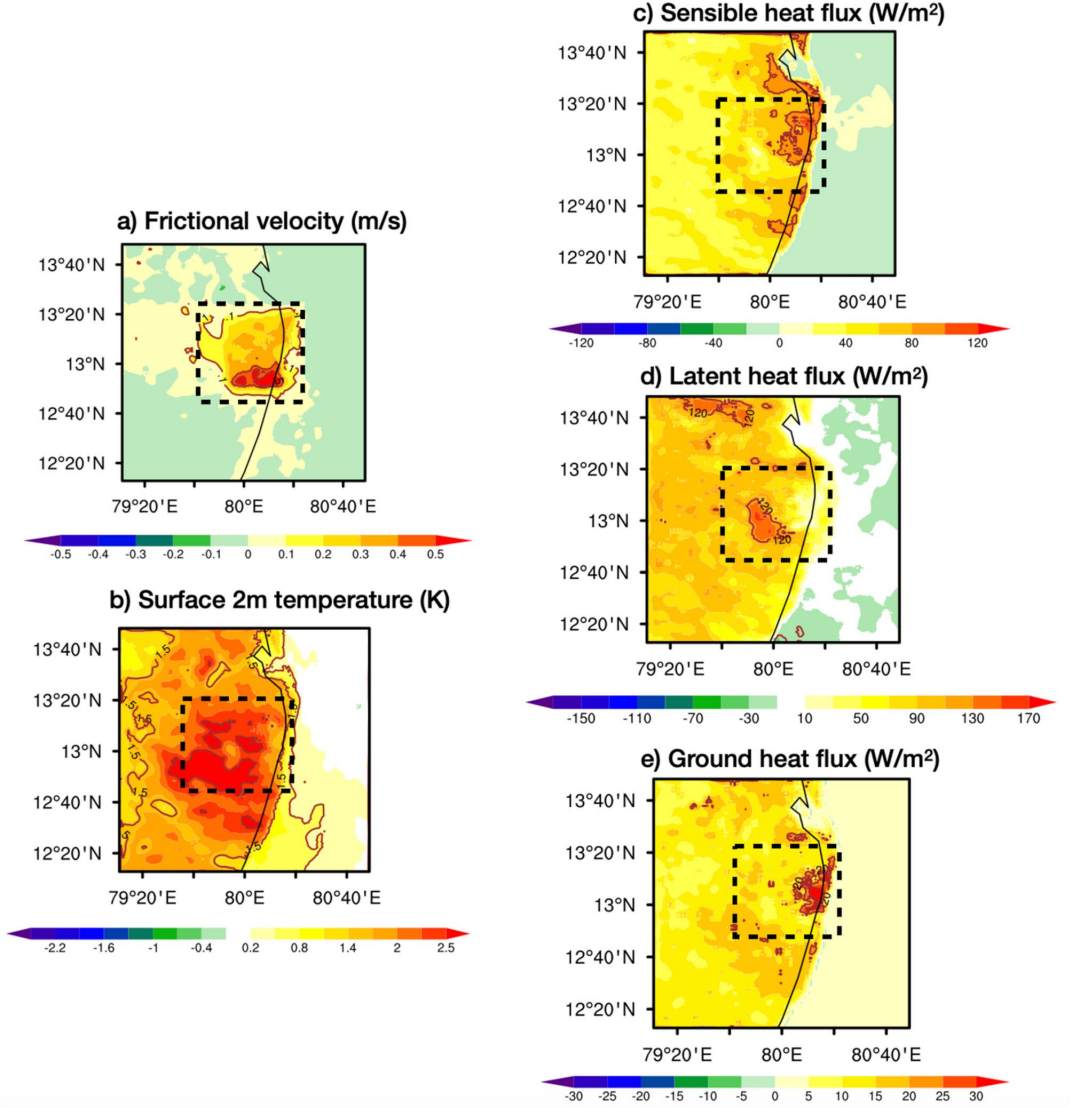
Differences in the LES simulation and physics ensemble before the heavy rainfall for (**a**) Frictional velocity (m/s), (**b**) 2-m surface temperature (K), (**c**) Sensible heat flux (W/m^2^), (**d**) Latent heat flux (W/m^2^), and (**e**) Ground heat flux (W/m^2^). Box shown in black/white dash line points Chennai city region.

To understand the role of urban induced turbulence UIT we computed differences in the LES and physics ensemble mean interpolated to the LES resolution (200 m) of frictional velocity, surface temperature, and surface fluxes. A higher frictional velocity (u*; >0.5 m/s) and elevated 2-meter temperature (T_2m_; >2 K) all over the Chennai city (Fig. 5a-b), especially its southern region, points to the significant contribution of urban environment to the UIT. Such contribution in turn enhances both mechanical and thermal mixing in the urban boundary layer. The complex surface roughness of urban areas contributes to the increase in u*, leading to greater momentum flux and intensified mechanical turbulence, while the Urban Heat Island (UHI) effect raises T_2m_, driving stronger thermal turbulence through buoyant convective currents. Together, these factors create a highly turbulent atmosphere over urban regions.

**Fig. 6:**
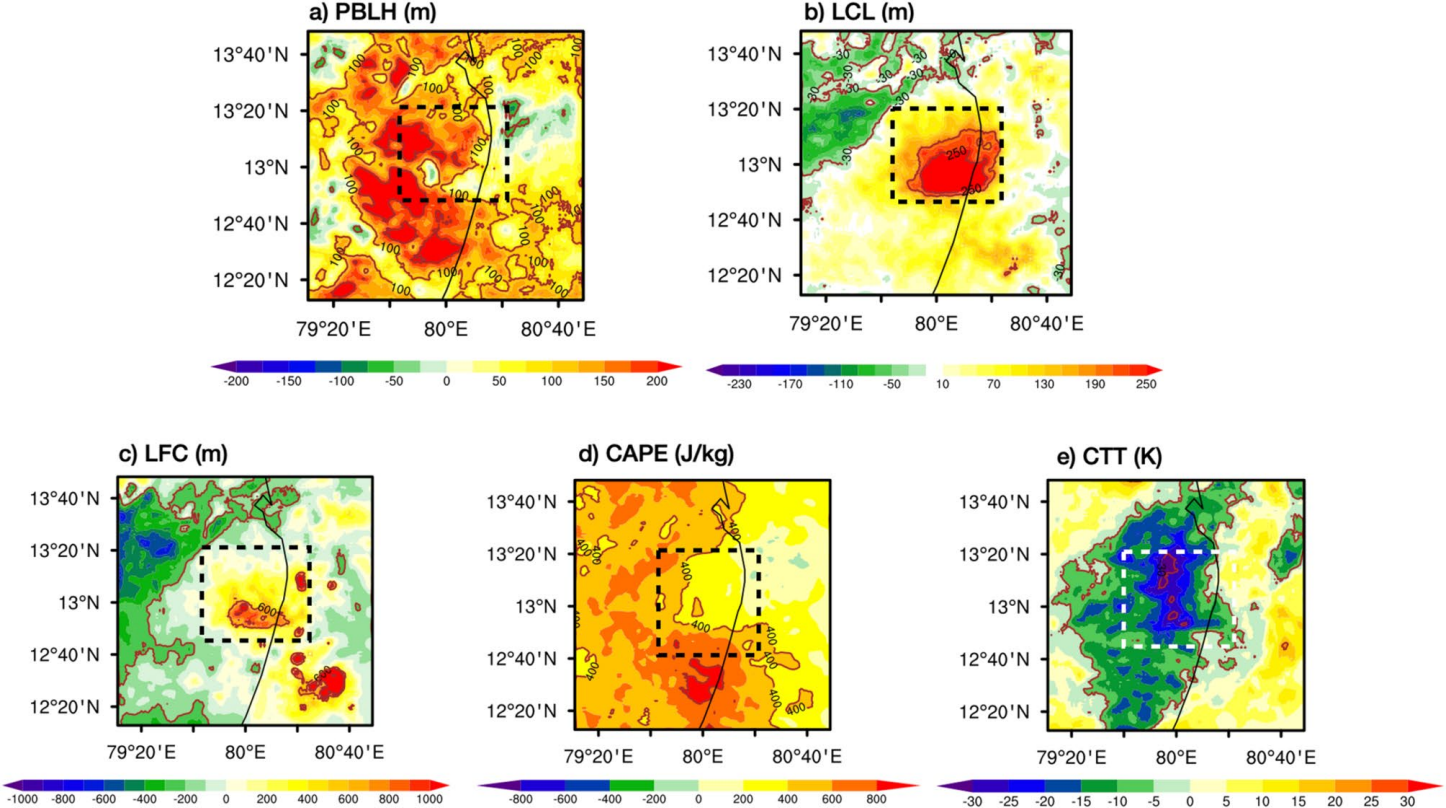
Differences in the LES simulation and physics ensemble before the heavy rainfall for (**a**) planetary boundary layer height (PBLH; m), (**b**) lifting condensation level (LCL; m), (**c**) level of free convection (LFC; m), (**d**) convective available potential energy (CAPE; J/kg) and during/after the heavy rainfall (**e**) cloud top temperatures (CTT; K). The contours show the differences. Box shown in black/white dash line points Chennai city region.

UIT and UHI strengthens turbulent heat fluxes and convective weather in the Urban boundary layer. To understand the role of turbulent fluxes (Fig. 5c-e) we computed differences in the LES and physics ensemble mean interpolated to the LES resolution (200 m) for sensible heat flux (SHFLX; W/m^2^), latent heat flux (LHFLX; W/m^2^), and ground heat flux (GHFLX; W/m^2^) before heavy rainfall. Over Chennai, higher frictional velocity supports increased SHFLX (Fig. 5c), which raises air temperature above urban surfaces, enhancing thermal turbulence and instability that can trigger convective activity. Elevated LHFLX (Fig. 5b) indicates more moisture being transferred from the surface to the atmosphere, fueling cloud formation and intensifying rainfall. A higher GHFLX (Fig. 5e) contributes to the warming of the surface during entire day and retaining ground heat even at night, sustaining the energy needed for continued convection. Together, these fluxes enhance the turbulent atmosphere over urban areas, creating conditions conducive to heavy rain events by promoting deep boundary layers (Fig. 6a), elevated lifting condensation levels (Fig. 6b), stronger levels of free convection (Fig. 6c), intense updrafts, higher convective available potential energy (CAPE; Fig. 6d), and deep cloud development as indicated by cloud top temperatures (CTT; Fig. 6e)..

## Discussions and summary

The Large-Eddy Simulation (LES) effectively captures the complex dynamics of urban-induced turbulence (UIT) and Urban Heat Island (UHI) effects, particularly in modeling heavy rainfall over urban areas such as Chennai. Unlike physics ensemble simulations, which could not resolve fine-scale urban atmospheric turbulence. LES’s higher resolution allows it to correctly represent localized turbulence and heat flux variations. This is crucial in urban environments where the complex interaction between built structures and atmospheric flows can significantly influence weather patterns. Previous studies, such as by^23,24^, have highlighted the importance of accurately modeling UHI effects in predicting urban weather. Recent studies^25,26^ has shown that LES is crucial in capturing small-scale processes that significantly impact urban meteorology, such as turbulent eddies and localized convective currents. These processes are vital in investigating and predicting extreme weather events, and our findings align with these understandings.

**Fig. 7:**
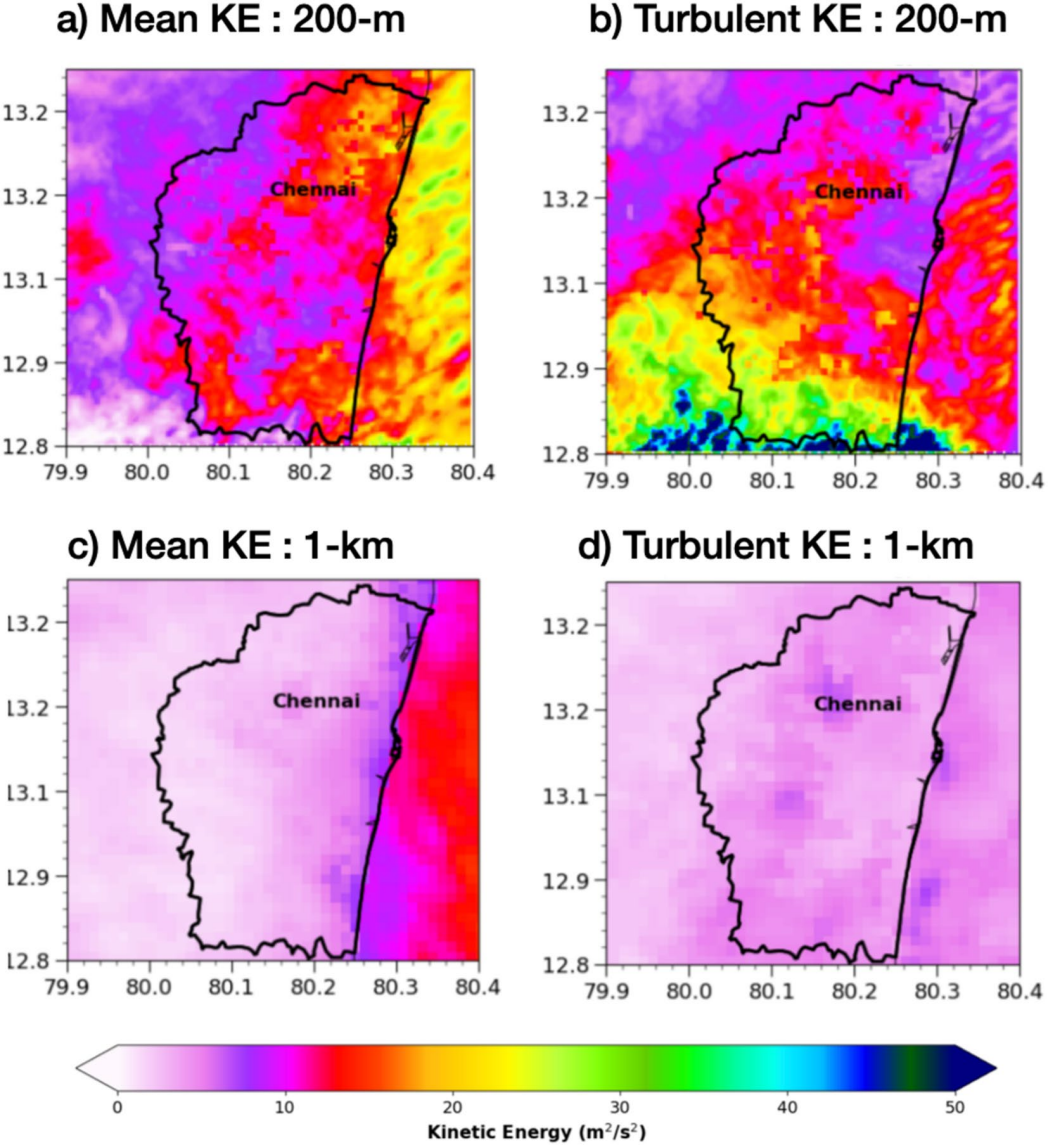
Spatial representation of mean and turbulent kinetic energy (MKE and TKE; m^2^/s^2^) shown for the 200-m LES and 1-km simulations before the heavy rainfall in the box shown in black/white dash line in Fig. 4. Chennai metropolitan boundary is shown in black contour.

To understand the different urban scales resolved by the LES simulation, mean and turbulent kinetic turbulent (MKE and TKE) was computed for Chennai region (Fig. 7) using Reynold`s decomposition (see methods section). MKE represents the energy associated with the organized, time-averaged wind flow, while TKE quantifies the energy in sub-grid, turbulent motions—key to understanding urban-scale eddies, mixing, and energy dissipation. In the LES, both MKE and especially TKE show large spatial variability, with higher KE and sharper gradients near the city, its downstream region, and its southeastern boundary, reflecting the model’s ability to resolve urban-induced turbulence across the Chennai region. In contrast, the 1-km simulation (bottom panels) displays much smoother, lower-amplitude MKE and almost featureless TKE fields, failing to capture the intense small-scale turbulence and variability generated by urban structures. Areas with higher TKE indicates to greater energy spectrum at smaller scales, as captured in the LES but under-resolved in the 1 km simulation. Earlier studies on urban^27^ showed that 1 km models still over-smooth convective up-drafts, while LES at 200–250 m captured realistic rain cell structure. This demonstrates the value of high-resolution LES for accurately capturing both the spatial distribution and spectral characteristics of urban turbulence during extreme weather events.

**Fig. 8:**
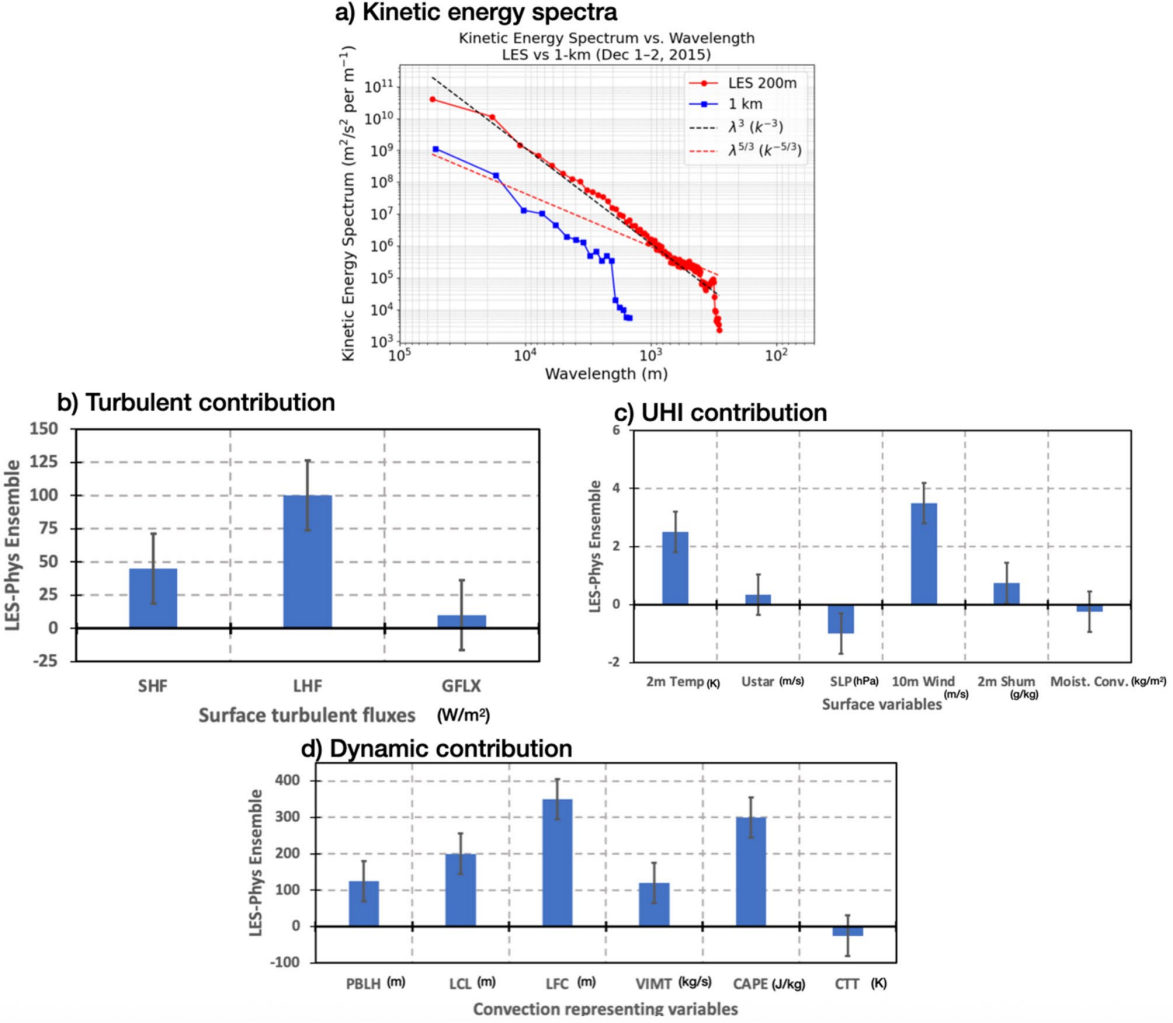
(**a**) Turbulent kinetic energy spectra shown for physics ensemble 1-km scale simulation (blue dots) and LES simulation (red dots) over the Chennai city domain during the heavy rain. Contributions from different parameters that represents (**b**) turbulence, (**c**) dynamics, and (**d**) urban heat island (UHI) over the Chennai city. In the bar plots error bars are shown for each contribution.

TKE spectra was computed for both the LES and the 1-km scale simulations over the Chennai regions (as shown in Fig. 7) during extreme weather conditions (Fig. 8a). The LES captures higher kinetic energy at smaller wavelengths, indicating its ability to resolve finer-scale atmospheric dynamics that are crucial in urban environments. This contrasts with the 1-km scale simulation, which shows a steeper drop-off in kinetic energy, highlighting its limitations in representing smaller-scale turbulence. The k^−5/3^ slope corresponds to the inertial subrange predicted by Kolmogorov’s theory for homogeneous and isotropic turbulence, where energy cascades efficiently from large to small scales without loss. The k^−3^ slope is characteristic of the enstrophy cascade in two-dimensional turbulence. The LES spectrum follows the k^−5/3^ and k^−3^ slopes over certain wavelength ranges, confirming the presence of realistic turbulent cascades in the simulation. The difference in energy cascades from larger scales to smaller scales suggests that LES is more effective in capturing the detailed interactions between urban structures and atmospheric flows, leading to more precise modeling of phenomena like UIT and UHI effects. This finding is consistent with studies^28,29^, which underscores the importance of high-resolution simulations in improving the accuracy of urban weather models, particularly for predicting heavy rainfall.

One of the key advantages of using LES over traditional physics ensemble simulations lies in its ability to better represent dynamic differences in surface conditions. The LES simulation reveals lower sea-level pressure, higher surface wind speeds, and concentrated moisture fluxes (Fig. 8b), which are critical factors for deep convective cloud formation and intense rainfall. These dynamic differences are driven by the enhanced turbulent mixing in urban areas (Fig. 8c), which is convincingly captured by LES through higher frictional velocity and elevated 2-meter temperature. These indicators of UIT and UHI (Fig. 8d), combined with increased sensible, latent, and ground heat fluxes, contribute to the development of a highly turbulent urban boundary layer. This, in turn, supports the formation of deeper clouds and more intense rainfall, a phenomenon that the physics ensemble simulations fail to represent accurately. Our findings suggest that LES is a superior tool for urban meteorological studies, particularly in predicting extreme weather events in highly urbanized regions. The outcomes of this research will inform urban planning and policymaking, helping to develop strategies for mitigating the impacts of extreme weather events. Additionally, this study will contribute to advancing the field of urban climatology by demonstrating the potential of LES as a valuable tool for improving weather forecasts and enhancing resilience in urban areas^30^.

The current study provides valuable understandings into the role of urbanization in exacerbating heavy rainfall, contributing to Sustainable Development Goals (SDGs) related to sustainable cities and communities (SDG 11) and climate action (SDG 13). By improving our understanding of how urban environments influence weather patterns, we can better prepare for and mitigate the impacts of extreme weather events in metropolitan areas. A key limitation of our study is its strong dependence on model resolution, parameterization choices, and no realistic representation of urban buildings, which can impact the representation of turbulence and cloud microphysics, especially in the urban context. The simplified land surface representations may not fully capture real-world urban complexity.

In future studies we would like to enhance the realism of these simulations by incorporating finer-scale resolutions of 5–10 meters, which would allow for more detailed modeling of individual buildings and their interactions with atmospheric processes. Such an approach, using a computationally scalable LES model, would provide even more accurate predictions of urban weather, aiding in the development of more resilient urban infrastructure and planning strategies.

### Data and methods

#### Model settings

The numerical simulation to analyze the flood event over Chennai was conducted using the Weather Research and Forecasting (WRF) model 3.9.1, configured for Large-Eddy Simulation (LES). The model domain covers the region around Chennai and its surrounding areas, as illustrated in the figure, capturing the topographical and geographical features crucial for accurate weather modeling. The simulation employed a nested grid approach with horizontal resolutions of 25 km, 5 km, 1 km, and an ultra-high resolution of 200 meters (supplementary Fig. S1). The LES mode was specifically utilized at the 200-meter resolution to explicitly simulate the planetary boundary layer (PBL) by applying a 1.5 TKE closure, allowing for detailed representation of turbulent processes in LES simulation. The simulation was initialized with reanalysis data from ERA5, ensuring that the large-scale atmospheric conditions were well represented.

The model settings for physics^31–39^ ensembles are similar to our earlier study on Chennai extreme rainfall^22^, which are provided in Table 1 except for LES simulation. Microphysics schemes used in the simulations include WSM6, Thompson, and MYNN, each offering different approaches to cloud and precipitation processes, which are critical for accurately capturing convective events. The MYNN, MYJ scheme^40,41^ PBL schemes were selected, with each scheme contributing to different representations of turbulence and mixing within the boundary layer. Cumulus parameterization was turned off in all domains to minimize the convection representation error^41^, which allows for the explicit simulation of convective processes without the need for parameterization. The time step for the model varied with resolution, with 90 seconds for the coarsest grid (25 km), scaling down to 5 seconds for the 200-meter grid, to maintain numerical stability and accuracy in the simulations.

**Table 1:** WRF model settings and initial condition dataset.

**Spatial horizontal grid (nested domains)**	25 km, 5 km, 1 km, 200-m
Cumulus parameterization	Off in all domains
Planetary boundary layer scheme	Mellor-Yamada-Janjic (MYJ), MYNN, Boulac, and LES setting (1.5 TKE closure)
Microphysics	WSM6
Land surface model	Noha land surface model
Urban scheme	SCUM
Surface physics	Monin-Obukhov-Janjic
Long and shortwave radiation	RRTM and Dudhia scheme
Vertical levels	30 terrain following coordinates
Land use dataset	MODIS Land use land cover dataset
Initial and boundary conditions	ERA5 6 hourly dataset

#### Urban canopy model

The WRF single-layer Urban Canopy Model (UCM)^32,42^ treats the city as an idealized, two-dimensional street canyon with infinite length and symmetric building rows. The formulation follows the original single-layer urban parameterization concepts^42^ and the coupled urban-heat-island experiments^43^, and it underpins subsequent urban-weather forecast applications^32^. Each time step UCM solves separate radiative budgets for the roof, wall and road facets including shadowing, multiple reflections and long-wave trapping and integrates a four-layer heat-conduction equation through those materials. Prognostic state variables such as roof, wall and road skin temperatures, canyon air temperature/humidity and canopy-level wind speed are advanced alongside the atmospheric grid, while momentum and heat exchange use roughness lengths and a drag term that scales with building density**.** UCM receives atmospheric forcing (wind, temperature, humidity, downward radiation) and returns updated fluxes and skin temperatures to the surface-layer, radiation and PBL schemes in the standard WRF physics order. Key geometric and thermal parameters building height, roughness lengths, sky-view factor, building fraction, heat capacities, conductivities, albedos and emissivity’s are read from the lookup table, while anthropogenic-heat and layer-thickness settings are set in default.

#### Numerical experiments

The experiment consisted of a total of 27 simulations, including various combinations of microphysics, PBL schemes, and horizontal resolutions to assess the sensitivity of the model to these parameters. The simulations were conducted over a 4-day period, starting from 29 November 00 UTC to 3 December 00 UTC, 2015, coinciding with an extreme weather event that resulted in significant flooding in Chennai^22^. The initial 24 hours of each simulation were treated as a spin-up period, allowing the model to stabilize and develop realistic atmospheric structures before the analysis period.

Three ensemble simulations were performed, each with different start times (00, 06, and 12 UTC) for each PBL schemes to account for uncertainties in the initial conditions and to provide a more robust estimate of the model’s performance. This ensemble approach is critical in weather forecasting and research, as it helps to understand the range of possible outcomes and the confidence in the model predictions. The high-resolution LES simulation at 200 meters was conducted with single realization. LES provides an in-depth analysis of the urban effects on local weather phenomena, including Urban Heat Island (UHI) and Urban Induced Turbulence (UIT), which are key factors influencing the development of deep convection and heavy rainfall in urban areas.

To evaluate model performance, simulations from the LES and the physics ensemble (10 members) are compared against observational data. For consistency and to highlight the benefits of high-resolution modeling, all model outputs including the 1 km physics ensemble are interpolated to the LES resolution (200 m). This allows for a direct comparison with observations and helps isolate the impact of resolution on model accuracy. Difference plots shown in Figures 2–4 and Figure 6 are generated using the ensemble mean of the physics simulations, interpolated to the LES grid, and then subtracted from the LES output. This approach emphasizes the spatial and structural improvements offered by LES over coarser models and provides a robust framework for assessing the influence of physical parameterizations and resolution on simulation fidelity.

#### Kinetic energy computation and its decomposition

The total kinetic energy (Total KE; m^2^/s^2^) of the surface flow, described by the velocity components U = (u, v), is calculated as shown in Equation (1), which sums the kinetic energy contributions from each velocity component^42^. By applying Reynolds’ decomposition^44,45^ to the velocity fields allows the decomposition of total kinetic energy, as in Equation (2), into two distinct components: the resolved mean kinetic energy (MKE), which is associated with the organized, time-averaged flow (the first term on the right-hand side), and the unresolved turbulent kinetic energy (TKE), which quantifies the energy contained in the fluctuating, sub grid-scale or turbulent motions (the second term).

1 $${\text{TotalKE}} = 1/2(u^{2} + v^{2} ) = 1/2{\text{U}}^{2}$$

2 $$1/2{\text{U}}^{2} = 1/2({\text{U}}^{2} ) + 1/2({\text{U}}^{2} )$$

This separation is fundamental for distinguishing the energetics of mean flows from those of turbulence, especially in urban and atmospheric simulations where both organized and turbulent structures play key roles in momentum and energy transport.

#### Precipitation observations

For this study, the high-resolution satellite dataset CHIRPS (Climate Hazards Group Infrared Precipitation with Station Data)^23,46^. Developed by the U.S. Geological Survey (USGS) in partnership with NASA (National Aeronautics and Space Administration) and NOAA (National Oceanic and Atmospheric Administration), CHIRPS provides an advanced, publicly accessible dataset for studying extreme precipitation events. It covers a broad geographic range from 50°N to 50°S with a high resolution of 5 km (25 km) for infrared-based precipitation data. Such high-resolution datasets are particularly beneficial for analyzing precipitation patterns, especially in regions like the foothills of the Himalayas^47^. The precipitation values in CHIRPS are derived using the CPC MORPHing technique and the TRMM 3B42 dataset and are subsequently refined with climatological data and gauge-based in-situ observations. This study utilizes CHIRPS data to examine extreme precipitation events from 1981 to 2015 in the Indian Himalayan region^48^.

## Supplementary Information

Supplementary Information.

## Data Availability

The Weather and Research Forecasting model Version 3.9.1 is available from the repository of National Center for Atmospheric Research (http://www2.mmm.ucar.edu/wrf/src/). ERA5 dataset was downloaded from the Copernicus 577 climate service which requires user registration (https://cds.climate.copernicus.eu/datasets/reanalysis-era5-pressure-levels?tab=overview). The OISST v2 data are obtained from the online website of the NOAA Earth System Research Laboratory’s Physical Sciences Division with registration (https://psl.noaa.gov/data/gridded/data.noaa.oisst.v2.highres.html). The Climate Hazards Group InfraRed Precipitation with Station data (CHIRPS) are available online (https://www.chc.ucsb.edu/data/chirps).
