# Supplementary material for: Urban-resolved scales amplifies torrential rainfall in coastal megacities
Source: Sci Rep. 2025 Nov 17;15:40170. doi: 10.1038/s41598-025-23906-y (PMC12623966; doi:10.1038/s41598-025-23906-y)
Supplement: Supplementary file 1 — Supplementary Information. [file 41598_2025_23906_MOESM1_ESM.docx]

Urban-Resolved Scales Amplifies Torrential Rainfall in Coastal Megacities

Konduru Rakesh Teja^1^, Rahul Bale^2, 3^, and Anu Gupta^4^

^1^Data Assimilation Research Team, RIKEN Center for Computational Science, Kobe, Japan

^2^Complex Phenomenon Unified Simulation Research Team, RIKEN Center for Computational Science, Kobe, Japan

^3^Graduate School of System Informatics, Kobe University, Japan.

^4^Graduate School of Information Science, University of Hyogo, Japan.

Corresponding author: Rakesh Teja Konduru ([rakeshtejak@gmail.com)](mailto:rakeshtejak@gmail.com))

Current address: Earth Observation Research Center, First Space Technology Directorate, Japan Aerospace Exploration Agency, Tsukuba Space Center, Sengen 1-1, Tsukuba, Ibaraki, Japan.

Supplementary material


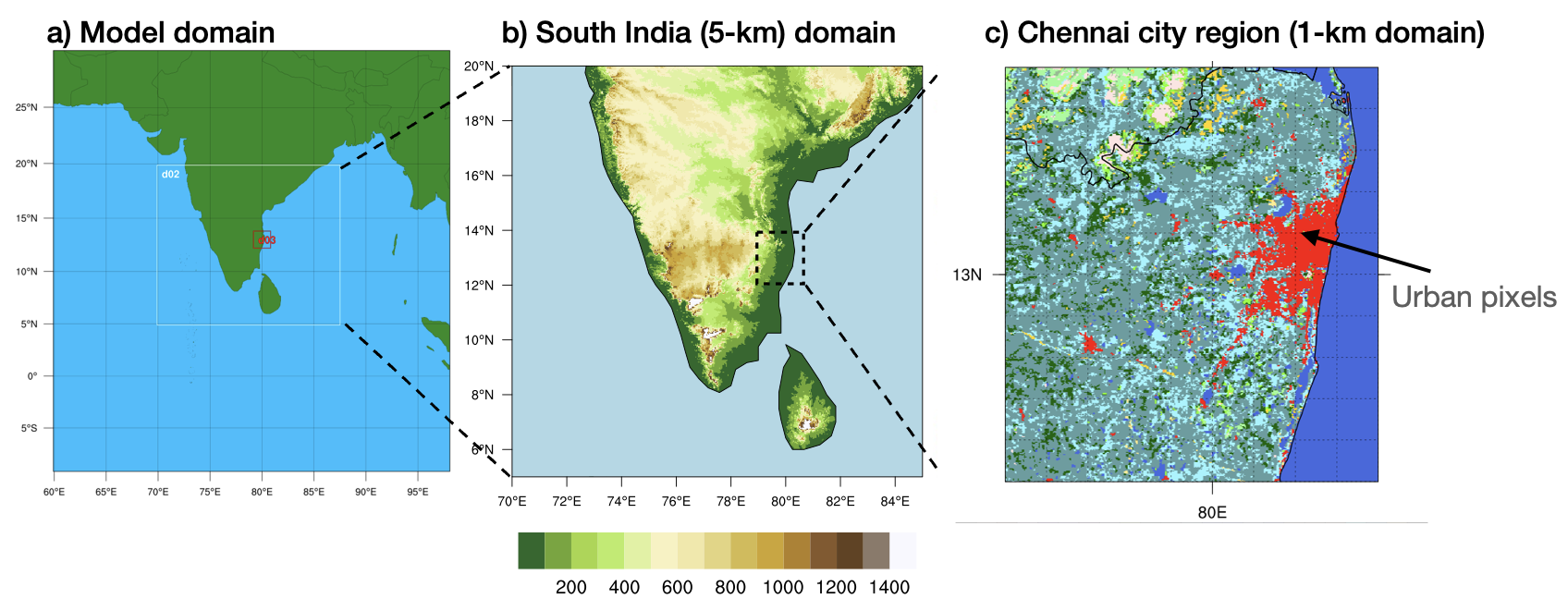


**Fig. S1** Model parent domains of a) 25-km, b) 5-km, and c) 1-km region over southeast India. Urban pixels of Chennai are pointed using arrow.


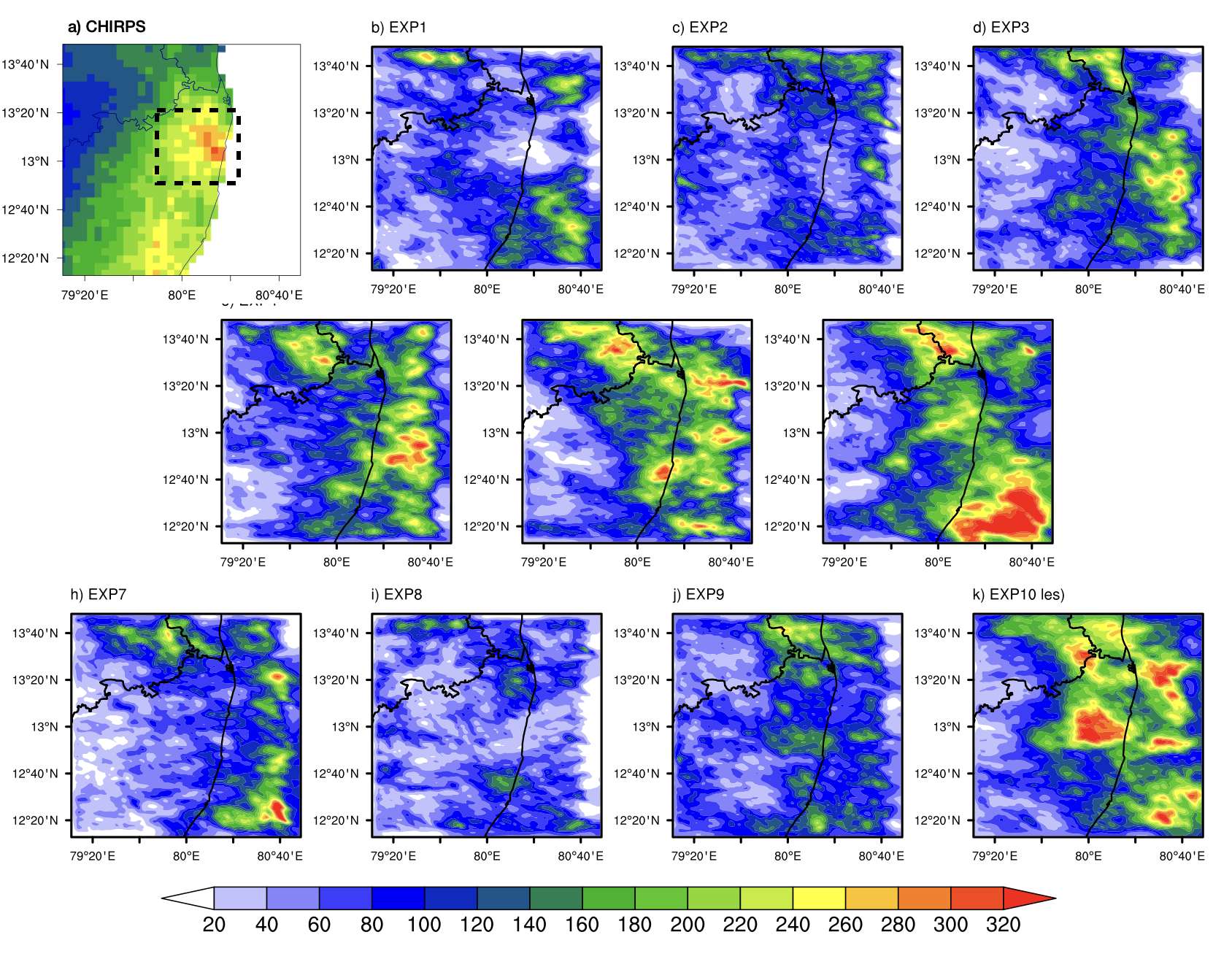


**Fig. S2** Accumulated precipitation over Chennai in a) CHIRPS observations, b-j) physics ensembles , and k) LES simulation. Dash box in (a) shows Chennai region.
